# COVID-19 pandemic: Impact on the management of patients with hepatocellular carcinoma at a tertiary care hospital

**DOI:** 10.1371/journal.pone.0256544

**Published:** 2021-08-26

**Authors:** Katharina Pomej, Bernhard Scheiner, Lukas Hartl, Lorenz Balcar, Tobias Meischl, Mattias Mandorfer, Thomas Reiberger, Christian Müller, Michael Trauner, Matthias Pinter

**Affiliations:** 1 Division of Gastroenterology and Hepatology, Department of Internal Medicine III, Medical University of Vienna, Vienna, Austria; 2 Liver Cancer (HCC) Study Group Vienna, Medical University of Vienna, Vienna, Austria; 3 Vienna Hepatic Hemodynamic Laboratory, Medical University of Vienna, Vienna, Austria; 4 Rare Liver Disease (RALID) Centre of the ERN RARE-LIVER, Medical University of Vienna, Vienna, Austria; 5 Christian Doppler Laboratory for Portal Hypertension and Liver Fibrosis, Medical University of Vienna, Vienna, Austria; Cliniques Universitaires Saint-Luc, BELGIUM

## Abstract

**Background:**

Patients with hepatocellular carcinoma (HCC) represent a vulnerable population potentially negatively affected by COVID-19-associated reallocation of healthcare resources. Here, we report the impact of COVID-19 on the management of HCC patients in a large tertiary care hospital.

**Methods:**

We retrospectively analyzed clinical data of HCC patients who presented at the Vienna General Hospital, between 01/DEC/2019 and 30/JUN/2020. We compared patient care before (period 1) and after (period 2) implementation of COVID-19-associated healthcare restrictions on 16/MAR/2020.

**Results:**

Of 126 patients, majority was male (n = 104, 83%) with a mean age of 66±11 years. Half of patients (n = 57, 45%) had impaired liver function (Child-Pugh stage B/C) and 91 (72%) had intermediate-advanced stage HCC (BCLC B-D). New treatment, was initiated in 68 (54%) patients. Number of new HCC diagnoses did not differ between the two periods (n = 14 vs. 14). While personal visits were reduced, an increase in teleconsultation was observed (period 2). Number of patients with visit delays (n = 31 (30%) vs. n = 10 (10%); p = 0.001) and imaging delays (n = 25 (25%) vs. n = 7 (7%); p = 0.001) was higher in period 2. Accordingly, a reduced number of patients was discussed in interdisciplinary tumor boards (lowest number in April (n = 24), compared to a median number of 57 patients during period 1). Median number of elective/non-elective admissions was not different between the periods. One patient contracted COVID-19 with lethal outcome.

**Conclusions:**

Changes in patient care included reduced personal contacts but increased telephone visits, and delays in diagnostic procedures. The effects on long-term outcome need to be determined.

## Introduction

Since December 2019, when the first cases of coronavirus disease 19 (COVID-19) were identified in Wuhan, China, severe acute respiratory syndrome-coronavirus-2 (SARS-CoV-2), a novel enveloped RNA betacoronavirus, rapidly spread across the globe, imposing numerous challenges on countries’ health systems and economies [1, 2]. After the world health organization (WHO) declared COVID-19 as a pandemic in early March 2020 [3], national health authorities and local governments, including Austria, were forced to implement various hygienic and physical distancing measures in order to stop the virus from spreading. Austria declared a total lockdown beginning from March 16, 2020, leading to the reallocation of resources especially in the healthcare sector. As of January 12, 2021, there have been 89 million confirmed cases of COVID-19 leading to 1.93 million deaths worldwide [4].

COVID-19-associated healthcare restrictions challenged the treatment of patients with chronic diseases and malignancies, including patients with HCC. HCC is among the top ten causes of cancer-related deaths in Austria with an age-adjusted mortality rate of around 4–5 per 100,000, and accounted for 4.5% of all cancer-related deaths in 2017 [5–8]. Patients with cancer are a high-risk group in the COVID-19 pandemic. Generally, they are more susceptible to any kind of infection due to an immunodeficient state caused by the underlying malignancy and anti-cancer treatments, such as systemic therapies and radiotherapy. Furthermore, as they require close follow-up, they are recalled to the hospitals more often, which further increases their risk of contracting SARS-CoV-2 infection [9]. While at the beginning of the pandemic, evidence from small retrospective studies suggested that cancer patients receiving anti-cancer treatments had an increased risk of mortality from COVID-19 than patients without anti-cancer therapies [9–11], this hypothesis was later on rejected, as mortality from COVID-19 seemed to be primarily determined by age, sex, comorbidities (e.g.: arterial hypertension, cardiovascular disease, diabetes mellitus), and ECOG PS in patients with active cancer [12–14]. Moreover, the risk of admission to intensive care unit (ICU) or the need for mechanical ventilation has been shown to be 3.5-fold higher in cancer patients [10, 15].

According to the recommendations of the European Association for the Study of the Liver (EASL) published during the COVID-19 crisis, management of patients with HCC should be maintained according to guidelines, including the administration of systemic treatments, the evaluation for liver transplantation, and the discussion of patients in multidisciplinary tumor boards [16]. Cancer surveillance for high-risk patients should be continued if possible, in order to minimize the risk of a future increase in cancer-related mortality [16].

The aim of this study was to evaluate the impact of the COVID-19 outbreak and accompanying governmental restrictions and reallocation of healthcare resources on the management of patients with HCC at Austria’s largest tertiary care hospital.

## Materials and methods

### Study design

We retrospectively included in- and outpatients with HCC who presented at the Vienna General Hospital/Medical University of Vienna between December 1, 2019 and June 30, 2020. Patients who were registered with the ICD diagnosis code “C22.0” were identified from the hospital’s electronic patient documentation system.

**Fig 1:**
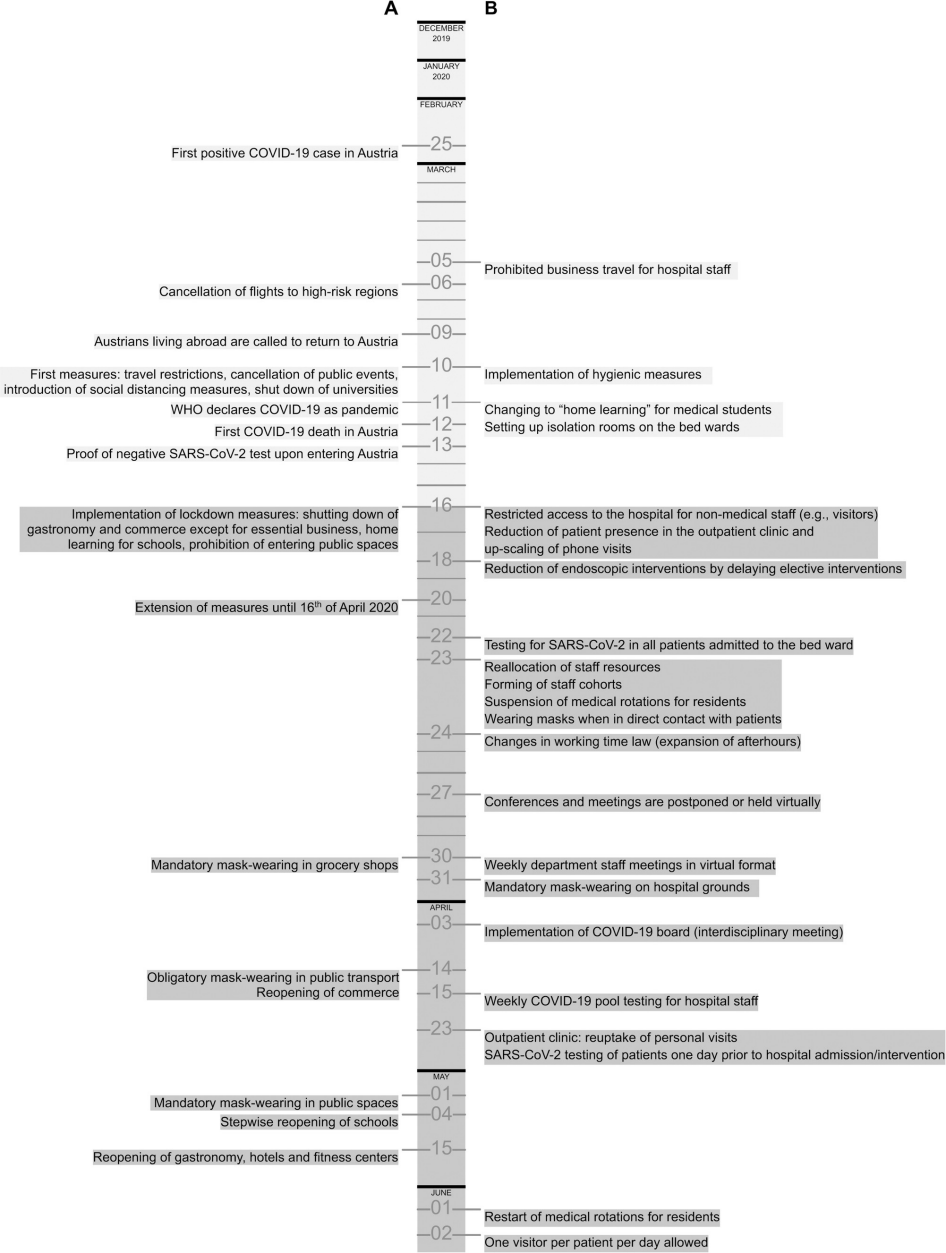
Timeline of COVID-19-associated healthcare restrictions. Measures implemented by A) the Austrian government and B) the Vienna General Hospital, Medical University of Vienna, Department of Internal Medicine, Division of Gastroenterology and Hepatology.

The observation time was divided in two periods, using March 16, 2020 as cut-off date, as it represents the day of the start of the hard lockdown in Austria. ***Period 1*** (December 1, 2019 –March 15, 2020) represents the time before COVID-19 measures were implemented (Fig 1) and ***period 2*** (March 16, 2020 –June 30, 2020) represents the time of COVID-19-associated governmental and healthcare restrictions.

In order to explore patients’ satisfaction and worries with the quality of care during COVID-19-related healthcare restrictions, we distributed a written questionnaire comprising 11 questions to patients with liver diseases in the Hepatology in- and outpatient service of the Medical University of Vienna starting in June 2020. All patients visiting the in- and outpatient clinic were asked to participate in the survey on a voluntary basis. In order to determine patient satisfaction with the quality of care at our department, a visual analogue scale (VAS), ranging from 0–10 points, was used.

Retrospective data analysis was approved by the local ethics committee of the Medical University of Vienna (#1690/2020 and #1461/2020) and performed according to the current version of the Helsinki Declaration (2013). Written informed consent was obtained from all patients completing the questionnaire.

### Patients and definitions

Eligible patients were adults (>18 years) diagnosed with HCC. Diagnosis of HCC was established either by histology or dynamic imaging (computed tomography [CT]/magnetic resonance imaging [MRI] scans) according to the European Association for the Study of the Liver (EASL) guidelines [7]. Patients with other liver tumors (e.g., cholangiocellular carcinoma), hepatic metastasis due to other primary malignancies, and one patient with insufficient records were excluded from this study. Patient data were collected from original patient records, including outpatient, discharge, and tumor board letters as well as radiological reports. Laboratory parameters were collected from laboratory reports from a network of hospitals in Vienna.

### Evaluation of patient visits, interventions, and imaging

All information on planned and performed visits, interventions, and imaging have been retrospectively collected from the clinical documentation system. ‘***Visits’*** were defined as outpatient and inpatient appointments as well as elective admissions to the ward of the Division of Gastroenterology & Hepatology. ‘***Interventions***’ included liver/tumor biopsy, hepatic surgery, microwave ablation [MWA], radiofrequency ablation [RFA], transarterial chemoembolization [TACE] and gastroscopy, liver related surgery, and liver biopsy. The term ‘***imaging***’ included routine imaging tests (e.g.: CT and MRI) for tumor response evaluation.

### Statistics

Statistical analyses of all data, including data from the questionnaire, were performed using IBM SPSS Statistics 26 (SPSS Inc., Armonk, New York, USA). Continuous variables were reported as mean ± standard deviation (SD) or median (IQR), and categorical variables were shown as numbers (n) and proportions (%) of patients. Comparisons of proportions and of continuous variables were performed by Chi-squared test and unpaired Student’s *t* test, respectively. For paired samples McNemar’s test and paired Student’s *t* test were applied. Kaplan-Meier method was used to calculate the median time of delays and survival curves. A two-sided p-value ≤0.05 was considered statistically significant.

A ‘***delay***’ was defined as difference ≥14 days between the planned and the actual date of visit/intervention/imaging. The difference of 14 days has been chosen, in order to incorporate the variation of appointments that can be expected in clinical routine due to statistical reasons. In order to compare the number of delays between the two periods, delays were assigned to period 1 and period 2 according to the date of planned visit/intervention/imaging, irrespective of possible cross-overs. Only patients who have presented to our clinic in both periods, were considered for the comparison (n = 102). For calculation of the duration of a delay, the date of planned visit/intervention/imaging was subtracted from the actual date of visit/intervention/imaging minus 14. Kaplan-Meier method was used to calculate the duration of delays. Patients who never showed up for the planned visit/intervention/imaging were censored at the last day of the observation period (March 15, 2020 for period 1 and June 30, 2020 for period 2). In patients with more than one delay in either visit, intervention, or imaging, we calculated the median time of delay in each individual patient, which was then used to calculate the median time of delay for the whole cohort of patients with a respective delay.

## Results

### Patient characteristics

**Fig 2:**
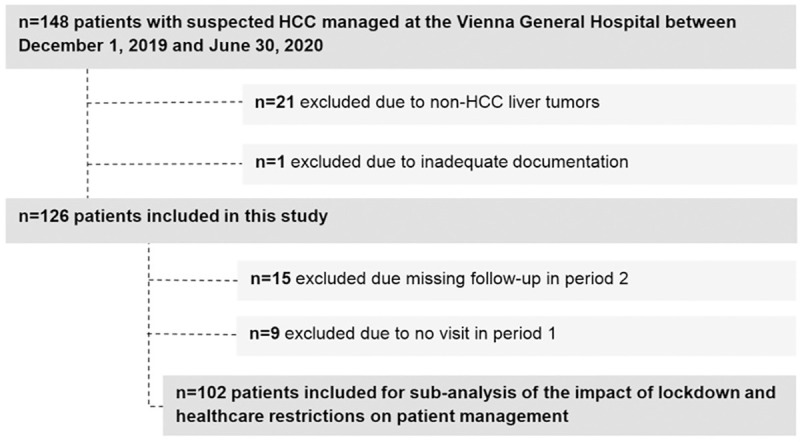
Patient flow-chart.

In total, 148 patients with suspected HCC presented at the Vienna General Hospital/Medical University of Vienna between December 1, 2019 and June 30, 2020. Of those, 22 patients were excluded from this study due to other liver tumors (n = 21) and inadequate documentation (n = 1) (Fig 2). Consequently, 126 patients were included in this study, of whom 24 patients were not considered for the comparison of the two periods due to missing follow-up in period 2 (n = 15) and no visit during period 1 (n = 9). Detailed patient characteristics are shown in Table 1. The majority of patients was male (n = 104, 83%) with a mean age of 66±11 years. One-hundred and three (82%) patients had cirrhosis. Viral hepatitis (38%) and alcoholic liver disease (30%) were the main etiologies. Sixty-nine patients had Child-Pugh stage A (55%) and the most frequent tumor stage was BCLC stage C (n = 47; 37%). Thirty-eight (30%) patients received systemic therapy at study inclusion. Forty-two (33%) patients were under observation after successful tumor treatment (surgery, loco-ablative therapy) while 43 (34%) patients were treatment-naïve at study inclusion. A new treatment was initiated in 68 (54%) patients during the observation period. Median time of follow-up was 5.4 (95%CI: 4.9–5.8) months.

**Table 1:** Patient characteristics.

		Number (%)
**Patients**		126 (100%)
**Age (years)**	Mean±SD	66±11
	Range	29–87
**Sex**	Male	104 (83%)
	Female	22 (17%)
**Cirrhosis**	Yes	103 (82%)
	No	23 (18%)
**Etiology**	ALD	38 (30%)
	Viral	48 (38%)
	Other	25 (20%)
	Unknown	15 (12%)
**Child-Pugh Classification**	A	69 (55%)
	B	47 (37%)
	C	10 (8%)
**ECOG PS**	0	96 (76%)
	≥1	30 (24%)
**Macrovascular invasion**	Yes	28 (22%)
	No	98 (78%)
**Extrahepatic metastases**	Yes	23 (18%)
	No	103 (82%)
**BCLC stage**	0	7 (6%)
	A	28 (22%)
	B	34 (27%)
	C	47 (37%)
	D	10 (8%)
**AFP (IU/mL)**	Median (IQR)	5.6 (2.5–214.3)
**Last HCC treatment before study inclusion**	Systemic Therapy	39 (31%)
	RFA/MWA/TACE	33 (26%)
	Resection/LTX	13 (10%)
	None	41 (33%)
**HCC treatment at study inclusion**	Systemic Therapy	38 (30%)
	RFA/MWA/TACE	3 (3%)
	Observation	42 (33%)
	None	43 (34%)
**New HCC treatment during observation period**	Yes	68 (54%)
	No	58 (46%)
**COPD**	Yes	14 (11%)
	No	112 (89%)
**Arterial hypertension**	Yes	72 (57%)
	No	54 (43%)
**Antihypertensive treatment** *	Yes	57 (45%)
	No	69 (55%)
**Statin therapy**	Yes	25 (20%)
	No	101 (80%)
**Coronary heart disease**	Yes	13 (10%)
	No	113 (90%)
**History of myocardial infarction**	Yes	4 (3%)
	No	122 (97%)
**Chronic kidney disease**	Yes	21 (17%)
	No	105 (83%)
**Smoking**	Yes	41 (33%)
	No	85 (67%)
**Diabetes mellitus**	NIDDM	36 (28%)
	IDDM	11 (9%)
	None	79 (63%)

* excluding Propranolol, Carvedilol, Furosemide, Aldactone.

**Abbreviations:** AFP, α-fetoprotein; ALD, alcoholic liver disease; BCLC, Barcelona clinic liver cancer; COPD, chronic obstructive pulmonary disease; ECOG PS, Eastern Cooperative Oncology Group performance status; IDDM, insulin-dependent diabetes mellitus; LTX, liver transplantation; MWA, microwave ablation; NIDDM, non-insulin dependent diabetes mellitus; RFA, radiofrequency ablation; TACE, transarterial chemoembolization.

### Prevalence of risk factors for COVID-19

Comorbidities (i.e., arterial hypertension, diabetes mellitus) have previously been established as risk factors for acquiring a severe COVID-19 infection and for mortality from COVID-19 [17, 18]. Chronic health conditions were frequently observed in our cohort (Table 1). The most common comorbidities apart from HCC were arterial hypertension (n = 72, 57%), followed by diabetes mellitus (n = 47, 37%), a history of smoking (n = 41, 33%), chronic kidney disease (n = 21, 17%), and chronic obstructive pulmonary disease (COPD) (n = 14, 11%).

### Impact of COVID-19-associated measures on diagnostic and therapeutic procedures

Of 126 patients included, 28 patients were newly diagnosed with HCC during the observation period. There was no difference in newly diagnosed HCC cases between period 1 (n = 14) and period 2 (n = 14). Baseline characteristics between patients diagnosed in period 1 and period 2 were not different (S1 Table).

In the subgroup of patients who had at least one hospital contact during both periods (n = 102), there was no significant difference in mean number of visits (in- and outpatient) between period 1 and period 2 (2.6±1.5 vs. 2.3±1.5; p = 0.157) (Table 2). In total, 40 (39%) patients had a delay in follow-up visits. Thirty-one (30%) patients had a visit delay in period 2 compared to only 10 (10%) patients in period 1 (p = 0.001). The median duration of visit delay was also numerically longer in period 2 (32 (95%CI: 7–58) days in period 2 vs. 21 (95%CI: 0–43) days in period 1). Interventions were performed in forty-seven (46%) patients of whom 5 (11%) patients had an intervention delay (3 gastroscopies and 2 microwave ablations) in period 2 compared to 0 intervention delays in period 1 (p = 0.063). The median time of intervention delay in period 2 was 22 days. Moreover, the number of patients with imaging delays was significantly higher in period 2 (period 1: n = 7 (7%) vs. period 2: n = 25 (25%); p = 0.001). The median duration of imaging delay was also longer in period 2 (34 (95%CI: 1–67) days in period 2 vs. 25 (95%CI: 0–61) days in period 1).

**Table 2:** Impact of COVID-19-related healthcare restrictions on patient management.

	Period 1 n = 102	Period 2 n = 102	p-value
**Number of visits per patient,** mean ± SD	2.6±1.5	2.3±1.5	0.157
**Patients with delay in visit,** n (%)	10 (10%)	31 (30%)	**0.001**
**Patients with delay of an intervention**, n (%) ^**a**^	0	5 (11%) *	0.063
**Patients with delay in imaging**, n (%) ^**b**^	7 (7%) *	25 (25%) *	**0.001**

^a^ Interventions performed in n = 47 patients.

^b^ Data available in n = 101 patients.

* Percentages calculated from respective sample sizes.

**Abbreviations:** COVID-19, coronavirus disease 2019; HCC, hepatocellular carcinoma.

### Impact of COVID-19-associated measures on patient contacts

**Fig 3:**
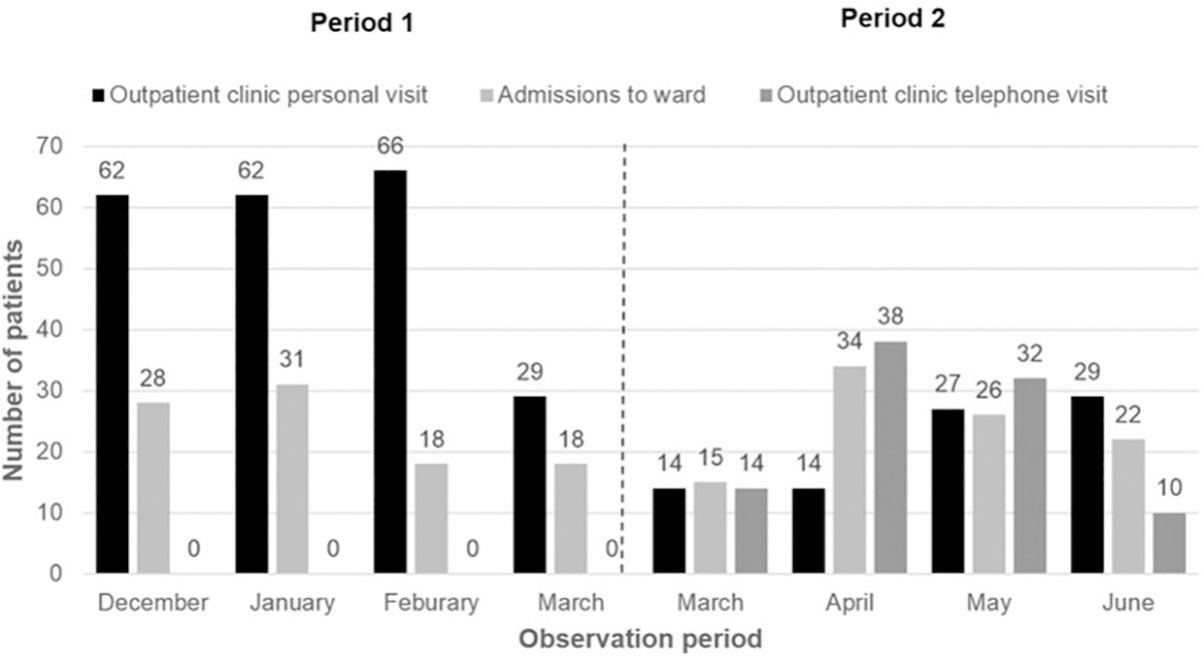
In- and outpatient frequencies at the department of Gastroenterology and Hepatology, including personal and telephone visits at the outpatient clinic and elective and non-elective admissions of patients with HCC to the ward.

**Fig 4:**
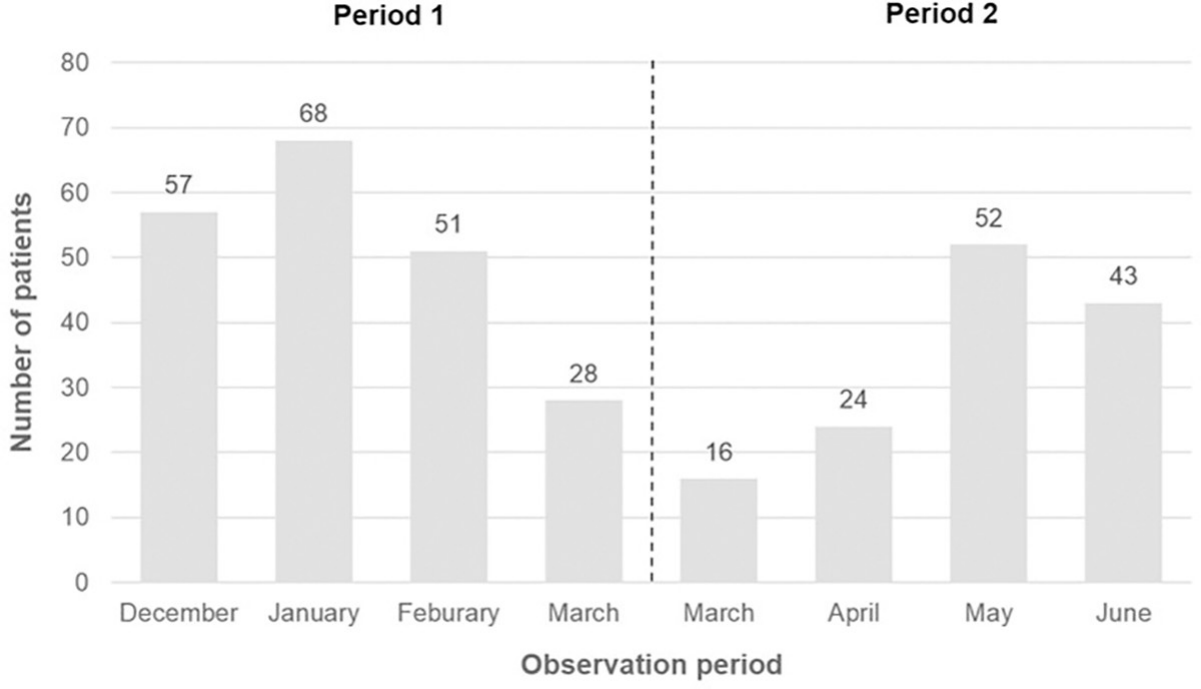
Patients discussed in weekly interdisciplinary tumor board.

There was a shift from personal visits towards telephone visits in the outpatient clinic starting with March 16, 2020 due to a policy change (Fig 3). While the median patient contact per month for personal visits was 62 patients during the first three months in period 1 (December-February), only a median of 22 patients were seen personally during the last three months of period 2 (April-June). Instead, we observed an increase in teleconsultation which peaked in April with a total of 38 patients (Fig 3). The median number of elective and non-elective admissions to the wards was 28 per month over the entire observational period (December 2019-June 2020), and not different between period 1 and period 2 (median, n = 23 and n = 24) (Fig 3). Finally, the number of patients discussed in the weekly interdisciplinary, hepatobiliary tumor board was lower during period 2, with the lowest number (24 patients) being discussed in April compared to a median number of 57 patients discussed per month during the first three months of period 1 (December-February) (Fig 4).

### SARS-CoV-2 testing and positivity

In total, 80 (64%) patients were tested for SARS-CoV-2 routinely (e.g., before imaging) in line with local COVID-19 regulations and had a total number of 248 SARS-CoV-2 tests (Table 3). In patients (n = 80) who received at least one SARS-CoV-2 test, the median number of tests per patient was 2 (IQR: 1–4). Only one patient (1.3%) was tested positive for SARS-CoV-2 and consequently developed COVID-19 pneumonia within the observation period. Initially, the patient was admitted to our inpatient ward for radiofrequency ablation of two HCC nodules on March 12, 2020. After discharge on the following day, the patient presented with dyspnea, fever, and hypotension at a different hospital on March 20, 2020. His initial PCR test for SARS-CoV-2 was negative. He was admitted to the ICU due to respiratory failure and sepsis. When his condition worsened, the patient was transferred to our hospital one week later, where he was tested positive for SARS-CoV-2 upon admission. Apart from HCC in non-alcoholic steatohepatitis (NASH)-related cirrhosis, the patient had arterial hypertension and diabetes mellitus type II as risk factors for the development of severe COVID-19. The patient’s condition gradually worsened. He developed a three-organ failure (lung, kidney, and liver) and died on May 6, 2020.

**Table 3:** SARS-CoV-2 testing.

		Total, n = 126
**Number of patients tested for SARS-CoV-2**	N, %	80 (64%)
**Total number of SARS-CoV-2 tests**	N	248
**Median number of SARS-CoV-2 tests of entire cohort**	Median (IQR), n = 126	1 (0–3)
**Median number of SARS-CoV-2 tests of tested patients only**	Median (IQR), n = 80	2 (1–4)
**Number of patients tested positive for SARS-CoV-2**	N, % n = 80	1 (1.3%)

**Abbreviations:** SARS-CoV-2, Severe Acute Respiratory Syndrome Coronavirus 2.

### Survival and follow-up of the whole cohort

Twenty-one of 126 patients (17%) died until June 30, 2020. Eight patients (38% of total deaths) died during period 1 and 13 patients (62% of total deaths) deceased during period 2. The majority of those patients (n = 20, 95%) died due to progression of the liver disease while one patient (5%) died from COVID-19.

### Patient perceptions on HCC care during initial phase of COVID-19 pandemic

Twenty-two patients with HCC completed the specifically designed questionnaire on patient satisfaction during COVID-19-associated healthcare restrictions. Although there was an increase in the number of visit, intervention and imaging delays after the implementation of restrictions (period 2), patient satisfaction with HCC care remained high as indicated by a visual analogue scale (VAS, ranging from 0–10) value of 8.9±1.4 before vs. 9.3±1.0 during COVID-19 measures. Only one (4.5%) patient reported dissatisfaction about the liver care provided during the pandemic due to reduced possibilities of contacting his general practitioner. Fear about negative implications of the pandemic on the underlying liver disease was present in approximately one third of patients with HCC (n = 6, 27%). None of the patients reported having problems with access to their medication, including anti-cancer treatments.

## Discussion

The first wave of the COVID-19 pandemic imposed significant challenges on healthcare systems and led to the reallocation of resources normally assigned to other diseases. The implementation of a state-wide hard lockdown in Austria involved numerous changes in standard clinical care activities for critically ill patients. In this study we provide real-world data on the implications on management of HCC patients during COVID-19-associated healthcare measures at a tertiary care hospital.

On 16^th^ of March 2020, a nationwide lockdown was introduced due to rising numbers of COVID-19 positive cases in Austria, rapidly leading to the flattening of the curve of the infection rate with SARS-CoV-2 in the general population [19]. Cancer patients are more susceptible to infections due to their systemic immunosuppressive state and were therefore thought to be at increased risk for acquiring SARS-CoV-2 especially in the beginning of the pandemic [10]. Studies from China reported a SARS-CoV-2 incidence among cancer patients of around 1% [9, 10]. In an Austrian cohort of patients with different cancer types, the infection rate was 0.4% (4/1016 patients), which was comparable to that of the general Austrian population and even lower when compared to patients without cancer [20]. We observed a similar rate in our cohort of patients with HCC, as only one of 80 tested patients (1.3%) contracted SARS-CoV-2; however, this patient died from COVID-19 pneumonia and multiorgan failure.

Several studies [13, 21] have demonstrated that patients with cancer appear to have an increased risk of mortality due to SARS-CoV-2 infection, with mortality rates ranging between 11% [22] and 28% [14]. In addition to well-known risk factors including age, sex, and concomitant metabolic diseases (e.g., arterial hypertension, diabetes mellitus), ECOG PS ≥2 and active cancer seem to be associated with worse outcomes from COVID-19 [13, 17, 23]. In our cohort, frequently observed risk factors were arterial hypertension (57%), diabetes mellitus (37%) and a history of smoking (33%). Furthermore, the patient with fatal COVID-19 outcome was 46 years old, male, and had active cancer. Moreover he was obese (BMI ≥30kg/m^2^ according to the current WHO guidelines [24]) and had two metabolic risk factors (arterial hypertension and diabetes mellitus), ECOG PS 0 and recently received RFA for active HCC.

Patients with advanced HCC are a very vulnerable and complex population as they usually suffer from concomitant underlying liver disease [25]. Thus, they require close follow-up and evaluation of their tumor status and adequate management of the underlying liver disease, resulting in frequent hospital visits [7, 25]. Implementation of remote care (i.e., telemedicine/telehealth) during pandemics is beneficial in keeping people safe by reducing the use of resources in healthcare, improving access to care, and minimizing the risk of direct transmission from person to person. At the Division of Gastroenterology and Hepatology, Medical University of Vienna, personal visits were reduced to a minimum and follow-up visits were predominantly conducted via telephone for the first two months after lockdown (Fig 3). Another COVID-19-associated healthcare measure was the change to virtual meetings in order to reduce personal contacts and the spreading of SARS-CoV-2 between departments within the hospital. As a consequence, the weekly hepatobiliary tumor board has been held in virtual format since the middle of March. Even though numbers of patients discussed in the tumor board were lower in the first two months after lockdown, these numbers went almost back to normal by the end of May. In general, video consultations and telemental health services were among other telemedicine services that were introduced during the COVID-19 pandemic [26]. However, many countries lack regulatory frameworks to properly authorize, integrate, and reimburse telemedicine services, and physicians’ concerns about technical and clinical quality of care, safety and privacy often remain [27, 28]. Nevertheless, we believe that telemedicine could be used as an alternative way of communicating with cancer patients who have a stable disease and are not requiring monthly face-to-face consultations, including the possibility to send drug prescriptions and referrals for upcoming imaging examinations by mail. Similarly, virtual conferences have proven of value as they represent a potential tool of communication in the future due to time effectiveness and simplicity.

As the pandemic progressed, international organizations including the American Association for the Study of Liver Disease (AASLD) [29] and the European Association for the Study of the Liver (EASL) [7] published guidelines on the management of patients with HCC and only few studies shared their real-life experiences and reported on the modifications made in the management algorithms of patients with HCC. While Iavarone and colleagues [30] only reported an increase in treatment delays of 2 months or longer in a small cohort of 42 HCC patients, Amaddeo et al. [31] observed a significant decrease of new diagnoses and treatment initiations in HCC patients. Notably in our study, the number of newly diagnosed HCC cases was similar before and after lockdown, suggesting that patients with a suspected malignancy still had access to rapid diagnosis and treatment initiation despite implementation of considerable healthcare restrictions. However, we observed significant differences in the number of patients with a delay in scheduled follow-up visits or imaging between the two periods. These differences can be explained by personal factors such as hospital avoidance due to the fear of contracting SARS-CoV-2, as well as institutional factors including the delay of imaging appointments and a general delay due to the implementation of SARS-CoV-2 testing before radiological interventions and admissions. Even after reducing patient occupancy of ward rooms to meet the COVID-19 hospital hygiene regulations, elective and non-elective admissions of patients with HCC did not significantly decline.

In general, patient satisfaction with HCC care remained high, even after implementation of COVID-19-related measures, although fear of negative impacts on the underlying liver disease was present in nearly one third of patients. While some European countries reported a massive impact of the COVID-19 pandemic on gastroenterology and hepatology departments, with 40% of the beds being repurposed for COVID-19 patients [32], we were able to largely sustain care of HCC patients at Austria’s largest tertiary care hospital, possibly due to the comparatively low incidence of COVID-19 in Austria during the first wave of the pandemic. However, we do believe that the inevitable effect of COVID-19 on liver diseases, including HCC, will be indirect and delayed, and thus, it may not be visible yet [33, 34].

We have to acknowledge some limitations for this study. First, we might have missed information on visits, delays, and risk factors for SARS-CoV-2 infection due to the retrospective design of the study and a possible lack of documentation. However, we thoroughly screened all outpatient documentation, discharge letters, and imaging reports and are confident that we can report at least all data that led to medical contact. Moreover, due to the limited sample size/duration of follow-up, this study was not sufficiently powered to assess the impact of delays, lower rates of patient presentations in interdisciplinary boards, and the shift from personal to telephone visits on patient survival.

In summary, we provide real-world data on the impact of SARS-CoV-2 on the management of HCC patients during the first wave of the pandemic in a large European tertiary care hospital. The incidence of SARS-CoV-2 infections was comparable with that of the general population. We observed a higher number of patients with visit and imaging delays after lockdown as well as lower numbers of patients discussed in tumor boards, while numbers of elective and non-elective admissions remained stable. Even though patient satisfaction with HCC care at our institution remained high, the long-term implications of the COVID-19 pandemic on the outcome of patients with HCC remain to be determined.

## Supporting information

S1 Table Baseline characteristics between period 1 and period 2 in patients with newly diagnosed hepatocellular carcinoma. (DOCX)

S1 File Questionnaire (DE). (PDF)

S2 File Questionnaire (EN). (DOCX)

## List of abbreviations

AFP: α-fetoprotein
BCLC: Barcelona clinic liver cancer
CI: confidence interval
COVID-19: coronavirus disease 19
CPS: Child-Pugh score
CRP: C-reactive protein
CT: computed tomography
EASL: European Association for the Study of the Liver
ECOG-PS: Eastern Cooperative Oncology Group performance status
HBV: hepatitis B virus
HCC: hepatocellular carcinoma
HCV: hepatitis C virus
ICU: intensive care unit
MRI: magnetic resonance imaging
MWA: microwave ablation
OS: overall survival
PCR: polymerase chain reaction
RFA: radiofrequency ablation
SARS-CoV-2: severe acute respiratory syndrome coronavirus 2
TACE: transarterial chemoembolization
VAS: visual analogue scale
WHO: world health organization
