# Supplementary material for: COVID-19 pandemic: Impact on the management of patients with hepatocellular carcinoma at a tertiary care hospital
Source: PLoS One. 2021 Aug 26;16(8):e0256544. doi: 10.1371/journal.pone.0256544 (PMC8389415; doi:10.1371/journal.pone.0256544)
Supplement: S1 Table — (DOCX) [file pone.0256544.s001.docx]

| **S1 Table. Baseline characteristics between period 1 and period 2 in patients with newly diagnosed hepatocellular carcinoma.** | | | |
| --- | --- | --- | --- |
|  | **Period 1**  **n=14** | **Period 2**  **n=14** | **p-value** |
|  | **(Number %) / Median (IQR)** | |  |
| **Sex**  Male  Female | 12 (86%)  2 (14%) | 12 (86%)  2 (14%) | 1.000 |
| **Child-Pugh Classification**  CPS A  CPS B  CPS C | 8 (57%)  5 (36%)  1 (7%) | 10 (72%)  3 (21%)  1 (7%) | 0.697 |
| **ECOG PS**  0  ≥1 | 10 (71%)  4 (29%) | 12 (86%)  2 (14%) | 0.648 |
| **Macrovascular invasion**  Yes  No | 3 (21%)  11 (79%) | 5 (36%)  9 (64%) | 0.678 |
| **Extrahepatic spread**  Yes  No | 2 (14%)  12 (86%) | 3 (21%)  11 (79%) | 1.000 |
| **BCLC stage**  A  B  C  D | 4 (29%)  3 (21%)  6 (43%)  1 (7%) | 3 (21.5%)  3 (21.5%)  7 (50%)  1 (7%) | 0.974 |
| **AFP** (IU/mL) | 5.7 (3.9-130) | 8.5 (1.7-64) | 0.376 |
| **Abbreviations:** AFP, α-fetoprotein; BCLC, Barcelona clinic liver cancer; CPS, Child-Pugh score; ECOG PS, Eastern Cooperative Oncology Group performance status; | | | |
