# Supplementary material for: COVID-19 pandemic: Impact on the management of patients with hepatocellular carcinoma at a tertiary care hospital
Source: PLoS One. 2021 Aug 26;16(8):e0256544. doi: 10.1371/journal.pone.0256544 (PMC8389415; doi:10.1371/journal.pone.0256544)
Supplement: S1 File — (PDF) [file pone.0256544.s002.pdf]

FRAGEBOGEN

<u>Medizinische Betreuungsqualität vor, während und nach der COVID-19-Pandemie</u>

Sehr geehrte Patientin, sehr geehrter Patient,
Die rezente COVID-19-Pandemie hat den Gesundheitssektor vor große Herausforderungen gestellt. Trotz der massiven Einschränkungen der Sozialkontakte bzw. des Zugangs zu Gesundheitseinrichtungen, sind bzw. waren wir bemüht eine möglichst gute medizinische Versorgung in Hinblick auf Ihre Lebererkrankung aufrecht zu erhalten. Auch nach Abklingen der Epidemie ersuchen wir Sie nochmals um Ihr Feedback, wie zufrieden Sie mit der Betreuungsqualität an unserer Ambulanz sind. Vielen Dank und alles Gute für Ihre Gesundheit,
Ihr ärztliches Behandlungs-Team der Abteilung für Gastroenterologie und Hepatologie.

1. **Alter in Jahren: _______________**
2. **Geschlecht** □ weiblich / □ männlich
3. **Fragebogen** □ zu Studienbeginn / □ nach 6 Monaten

Bitte beurteilen Sie anhand der folgenden Skalen ob Sie einer/m FreundIn / Bekannten eine Betreuung an unserer Ambulanz empfehlen würden (0=keine Empfehlung, 10=starke Empfehlung). KREISEN sie bitte Ihre Antwort ein!

0 1 2 3 4 5 6 7 8 9 10
☹ sicher nicht auf jeden Fall ☺

4. **Wie zufrieden waren Sie mit der Betreuungsqualität an unserer Ambulanz <u>VOR</u> der COVID-19-Pandemie?**

0 1 2 3 4 5 6 7 8 9 10
☹ sicher nicht auf jeden Fall ☺

5. **Wie zufrieden waren Sie mit der Betreuungsqualität an unserer Ambulanz <u>WÄHREND</u> der COVID-19-Pandemie?**

0 1 2 3 4 5 6 7 8 9 10
☹ sicher nicht auf jeden Fall ☺

6. **Wie zufrieden sind Sie mit der Betreuungsqualität an unserer Ambulanz <u>NACH</u> der COVID-19-Pandemie?**

0 1 2 3 4 5 6 7 8 9 10
☹ sicher nicht auf jeden Fall ☺

Herr Assoc.Prof. Priv.–Doz. Dr. Thomas Reiberger

7. **Für wie viele persönliche Besprechungstermine sind Sie während der COVID-19-Pandemie an die Gastroenterologische Ambulanz, Leitstelle 7i, gekommen?**

☐ Ich hatte (bisher) keine persönlichen Besprechungstermine in der Ambulanz.

☐ (Bisher) 1-2 persönliche Besprechungstermine.

☐ (Bisher) mehr als 2 persönliche Besprechungstermine.

8. **Wie oft hatten Sie während der COVID-19-Pandemie telefonischen oder E-Mail-Kontakt mit ÄrztInnen der Abteilung für Gastroenterologie und Hepatologie?**

☐ Ich hatte (bisher) keinen telefonischen / E-Mail-Kontakt.

☐ (Bisher) 1-2 telefonische / E-Mail Besprechungen.

☐ (Bisher) mehr als 2 telefonische / E-Mail Besprechungen.

9. **Mussten Sie während der COVID-19-Pandemie akut medizinische Hilfe in Anspruch nehmen? (Mehrfachnennungen möglich)**

☐ Nein, bisher nicht.

☐ Ja, ich musste akut meinen Hausarzt kontaktieren / aufsuchen.

☐ Ja, ich musste akut einen Facharzt / eine Spitalsambulanz kontaktieren / aufsuchen.

☐ Ja, ich musste akut die Rettung / den Ärztenotdienst kontaktieren.

10. **Hatten Sie während der COVID-19-Pandemie größere Schwierigkeiten ärztlichen Rat einzuholen als davor?**

☐ Nein, der Kontakt mit meinen betreuenden ÄrztInnen war komplikationslos.

☐ Ja, der Kontakt mit meinen betreuenden ÄrztInnen war mühsam, aber möglich.

☐ Ja, der Kontakt mit meinen betreuenden ÄrztInnen war kaum herzustellen, bzw. quasi unmöglich.

Herr Assoc.Prof. Priv.–Doz. Dr. Thomas Reiberger

11. **Hatten Sie <u>während der COVID-19-Pandemie</u> größere Schwierigkeiten Ihre Medikamente zu bekommen als davor?**

☐ Nein, ich habe meine Rezepte und Medikamente so wie immer erhalten.

☐ Ja, es war deutlich mühsamer / unmöglich die Rezepte für meine Dauermedikation zu erhalten.

☐ Ja, es gab Lieferengpässe meiner Dauermedikation.

12. **Fühlen Sie sich ausreichend über die möglichen Folgen einer SARS-CoV-2-Infektion auf Ihre Lebererkrankung informiert? (Mehrfachnennungen möglich)**

☐ Nein, ich fühl(t)e mich nicht ausreichend gut informiert.

☐ Ja, ich empfinde (empfand) die Aufklärung über Printmedien (Zeitungen, etc.), Internet und Fernsehen als ausreichend.

☐ Ja, ich wurde durch meine behandelnden ÄrztInnen ausreichend aufgeklärt.

13. **Haben Sie <u>während der COVID-19-Pandemie</u> mehr Bedenken über mögliche negative Auswirkungen auf Ihre Lebererkrankung als davor?**

☐ Nein.

☐ Ja.

14. **Fühlen Sie sich <u>während der COVID-19-Pandemie</u> medizinisch schlechter versorgt als davor?**

☐ Nein, ich empfinde (empfand) die Versorgungsqualität als unverändert.

☐ Ja, meine medizinische Versorgung ist / war während der COVID-19-Pandemie schlechter als davor.

15. **Was möchten Sie uns sonst noch mitteilen?**

____

____

Herr Assoc.Prof. Priv.–Doz. Dr. Thomas Reiberger

Vielen Dank für die Beantwortung der Fragen !!
