# Supplementary material for: COVID-19 pandemic: Impact on the management of patients with hepatocellular carcinoma at a tertiary care hospital
Source: PLoS One. 2021 Aug 26;16(8):e0256544. doi: 10.1371/journal.pone.0256544 (PMC8389415; doi:10.1371/journal.pone.0256544)
Supplement: S2 File — (DOCX) [file pone.0256544.s003.docx]

QUESTIONNAIRE

**Age in years**

**Gender: male / female**

**Questionnaire: at start of study / after 6 months**

**Would you recommend treatment in our Hepatology clinic to a friend? scale 0-10**

1. How satisfied were you with your treatment at the Vienna General Hospital **before** COVID-19-related healthcare restrictions on a scale of 0-10?
2. How satisfied were you with your treatment at the Vienna General Hospital **during** COVID-19-related healthcare restrictions on a scale of 0-10?
3. How satisfied are you with your treatment at the Vienna General Hospital **after** COVID_19-related healthcare restrictions on a scale of 0-10?
4. How many “in-person” visits did you attend at the Vienna General Hospital during the COVID-19-pandemic? 0=none, 1=1-2, 2>2
5. How many times did you have contact via telephone or email with doctors of the Hepatology Clinic during the COVID-19 pandemic? 0=none, 1=1-2, 2>2
6. Did you acutely need medical help during the COVID-19 pandemic? yes/no
7. Did you encounter more difficulties in searching medical advice during the COVID-19 pandemic than before? yes/no
8. Did you encounter more difficulties in getting your medication during the COVID-19 pandemic than before? yes/no
9. Do you feel sufficiently informed about the consequences of a SARS-CoV-2-infection on your liver disease? yes/no
10. Do you have concerns about negative effects on your liver disease during the COVID-19 pandemic? yes/no
11. Do you have the feeling that your medical treatment and management is worse during the COVID-19 pandemic than before? yes/no
